# Injury Patterns and Predictors of Delays to Obtaining Computed Tomography in the North Region of Cameroon

**DOI:** 10.1155/rrp/8575274

**Published:** 2026-04-24

**Authors:** Joshua Tambe, Yannick Onana, Nicholas Tendongfor, Marie-José Essi, S. Ariane Christie, Alain Chichom-Mefire

**Affiliations:** ^1^ Department of Internal Medicine and Pediatrics, University of Buea, Buea, Cameroon, ubuea.cm; ^2^ Department of Medical Imaging, University of Garoua, Garoua, Cameroon; ^3^ Department of Public Health and Hygiene, University of Buea, Buea, Cameroon, ubuea.cm; ^4^ Department of Public Health, The University of Yaoundé I, Yaoundé, Cameroon, uy1.uninet.cm; ^5^ Department of Surgery, University of California in Los Angeles, Los Angeles, California, USA; ^6^ Department of Surgery and Specialties, University of Buea, Buea, Cameroon, ubuea.cm

## Abstract

**Introduction:**

Injuries constitute a global public health problem, with a higher disease burden in low‐ and middle‐income countries where disability and mortality are significantly high. Accessibility concerns nevertheless persist regarding computed tomography (CT) utilization, the imaging workhorse for the assessment of most injuries. This study aimed to contribute to understanding injury patterns in Cameroon and to assess delays in obtaining CT in a low human development index setting.

**Methods:**

A cross‐sectional hospital‐based study was conducted from July 2023 to December 2024 at a university‐affiliated referral hospital in the North Region of Cameroon. Data were collected from all patients aged 18 years and above who were referred with moderate to severe injuries after consent was obtained. Logistic regression modeling was used to identify predictors of delays to CT utilization, and the threshold for statistical significance was set at 0.05.

**Results:**

A total of 511 participants were enrolled, with a sex ratio of 1.8:1 and a mean (SD) age of 30.6 ± 6.4 years. Road traffic injuries (RTIs) accounted for 69.9% (275/393) of injuries, followed by stab wounds (11.9%; 47/393). Motorbike use was reported in 52.4% (144/275) of the patients and was also the primary means of transportation to the referral hospital (55.3%; 213/385). Overall, extremity fractures were the most common lesions (49.6%; 192/387). Predictors of delays to obtaining CT were age < 40 years (*a*OR = 7.6, 95%CI: 2.4–23.9, and *p* = 0.001), male sex (*a*OR = 4.9, 95%CI: 2.3–10.7, and *p* < 0.001), reporting financial difficulties (*a*OR = 13.8, 95%CI: 4.9–38.9, and *p* < 0.001), and sustaining an RTI (*a*OR = 3.9, 95%CI: 1.9–7.9, and *p* < 0.001).

**Conclusion:**

RTIs are the predominant injury mechanism in the North Region of Cameroon, involving mostly motorbikes and young males for whom CT access is a challenge. Reinforcing policies that ensure the safety of all road users and that facilitate access to CT imaging would improve health outcomes.

## 1. Introduction

Injuries constitute a considerable disease burden worldwide, and over 90% of injury‐related deaths occur in low‐ and middle‐income countries [1, 2]. Although technological advances in healthcare have led to improved outcomes for patients with injuries in recent years, the burden of injuries has continued to rise in low‐ and middle‐income countries [3]. This rise in the burden of injuries is attributed to a combination of factors, which include the state of road infrastructures and vehicles, the attitude of road users, unsafe housing, living and working conditions, alcohol consumption, illicit drug use, and an increase in interpersonal conflicts [4–6].

Available data show that in Cameroon, injuries are a frequent reason for consultation and admission at hospital emergency departments (EDs), with the most frequent mechanisms being road traffic injuries (RTIs), assaults, and domestic injuries [7–9]. Medical imaging plays an important role in the management of patients with injuries by providing accurate and timely diagnoses to support appropriate and timely decision‐making for treatment. Modalities often used in trauma imaging include conventional radiography, ultrasonography (extended Focused Assessment with Sonography for Trauma [eFAST]), and multislice computed tomography (CT). However, these modalities are not equitably distributed across the national territory, and even when available, access remains a challenge, especially for CT. CT remains quite expensive yet vital for the assessment of moderate to severe head and torso injuries [9–11]. Timely access to imaging in patients with injuries is crucial, as delays have been reportedly associated with an increase in hospital length‐of‐stay and early mortality [12, 13].

The North Region of Cameroon has one of the lowest human development index (HDI) values across the country [14]. This low HDI is characterized by poverty, lack of access to quality healthcare and education, growing insecurity due to armed conflicts, and some natural disasters such as floods and droughts. Despite reports on injury mechanisms and the role of imaging in outcomes in other parts of the country [7, 8, 14], very little is known in this region. We, therefore, aspired to contribute to the understanding of the epidemiology of injuries in Cameroon through this study, which sought to describe the patterns and assess access to CT, an imaging workhorse for most emergencies, including injuries.

## 2. Materials and Methods

### 2.1. Study Design and Setting

A hospital‐based cross‐sectional study was conducted at the ED and surgical wards of Garoua Regional Hospital, a university‐affiliated intermediate‐level referral hospital for the North Region of Cameroon, from July 2023 to December 2024. This hospital, with over 300 beds, has a modern Radiology department equipped with state‐of‐the‐art multislice CT, digital radiography, and a high‐resolution ultrasound scanner. Other units include operating theatres, wards for admissions, and other outpatient, general, and specialized medical services. The staff includes hospitalists and academics who participate in patient care and in the training of medical students and other allied healthcare professionals. Garoua is the chief town of Cameroon’s North Region and hosts the region’s pioneer referral hospital. The main means of transportation within the city are commercial motorbikes, with few taxis and mostly private cars serving to reach neighboring towns and villages.

### 2.2. Ethics

This study was conducted in accordance with the ethical principles of the Declaration of Helsinki. Ethical clearance was obtained from the Institutional Review Board of the Faculty of Health Sciences, University of Buea (reference: 2023/2058–03/UB/SG/IRB/FHS). Administrative authorization to collect research data was further issued by the management of Garoua Regional Hospital (reference No.: 1131/23/L/HRG/CM). Informed consent was obtained from the patient or a legal proxy, and, due to variations in literacy, it was provided either in writing or verbally. All data collection forms were anonymized and retained by the principal investigator.

### 2.3. Respondents, Data Collection, and Analysis

Data were collected from all patients 18 years old and above with moderate to severe injuries by a trained research assistant. Injury severity classification was based on Injury Severity Scores (ISSs), with scores of 9–15 defined as moderate and scores > 15 as severe [15, 16]. For patients who could not provide the information due to their clinical state, information was obtained from a proxy, usually the caregiver. Data were collected using a pretested, structured data collection form that contained mostly closed‐ended questions, with a few open‐ended questions. The data form contained information on sociodemographic characteristics of the patient (age, sex, level of education, marital status, occupation, and place of residence), nature of injury and mechanism, type of vehicle involved (for RTIs), means of transportation to hospital, anatomic location of the injury (head, chest, abdomen, upper limbs, lower limbs, and multiple regions), clinical parameters including the Glasgow Coma Scale on arrival, respiratory rate, blood pressure, Abbreviated Injury Score (AIS), ISS, and the type of imaging studies requested and performed. The estimated time to hospital arrival following the injury and the time to obtain a CT following the order placement for the patients for whom it was placed were also assessed. Not all patients with injuries required a CT, and the placement of the order implied it was needed, and this item was assessed only for the patients for whom a CT order was placed.

The data were transcribed onto a Microsoft Excel spreadsheet and imported into Stata/MP 17.0 (StataCorp, Texas, USA) for statistical analysis. Continuous and categorical variables were summarized and presented as the mean (± standard deviation), counts, and percentages, respectively. The main outcome variable was delay in obtaining a CT, defined in this study as a CT obtained after 24 h or never obtained after the order was placed. Despite the golden hour recommendation for time to CT, this definition was practical, given that moderate‐to‐severe injuries were considered as a single category, and reports on the association between in‐patient mortality and a one‐hour time to obtain CT have been conflicting [16–20]. In routine clinical practice, this definition proved to be practical. Independent associations between this outcome variable and selected independent variables of interest were assessed using chi‐squared or Fisher’s exact tests. A multivariable logistic regression analysis was performed to determine factors associated with delays in obtaining CT. Model covariates were selected based on the literature and statistical criteria. A stepwise approach was used to build the model, with entry and exit probabilities set at 0.05 and 0.25, respectively. Model fit was determined using Akaike’s and Bayesian Information Criteria, and the model with the lowest values for these coefficients was retained. All statistical tests were two‐tailed, and the significance threshold was set at 0.05.

## 3. Results

### 3.1. Characteristics of Respondents

**FIGURE 1:**
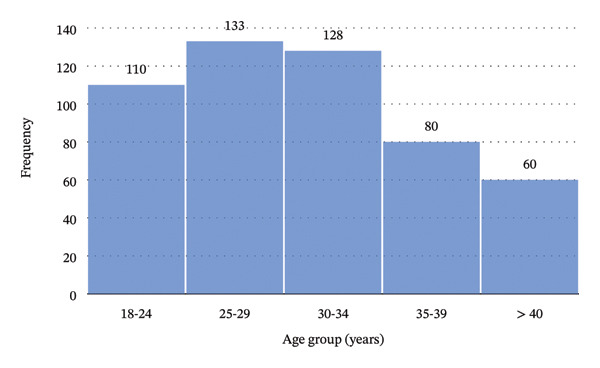
Age group distribution of the respondents.

Data were collected from 511 patients, of whom 328 (64.19%) were male, giving a sex ratio of 1.8:1. The mean (±standard deviation) age was 30.60 (±6.39) years, with a range of 18–44 years. There was no significant difference in the mean age between males and females. Figure 1 illustrates the age distribution of the respondents, whilst Table 1 summarizes their characteristics.

**TABLE 1:** Characteristics of the respondents.

Variable	Mean (±SD^∗^) or frequency (%)
Age (years; *N* = 511)	30.60 (±6.39)
Sex (*N* = 511)	
Male	328 (64.19)
Female	183 (35.81)
Level of education (*N* = 401)	
Completed primary school	20 (4.99)
Completed secondary school	51 (12.72)
Completed high school	200 (49.88)
Earned a university degree	130 (32.42)
Occupation (*N* = 465)	
Casual laborer	46 (9.89)
Civil servant	85 (8.28)
Motorbike rider	34 (7.31)
Housewife	31 (6.67)
Shepherd	37 (7.96)
Farmer	32 (6.88)
Student	18 (3.87)

^∗^SD: standard deviation.

### 3.2. Injury Mechanisms

There were 275 reported cases of RTIs out of 393 (69.97%) of which 144 (52.36%) were reportedly due to motorbike accidents. The main means of transporting injured patients from the injury site to the referral hospital was motorbikes in 55.32% of the cases. Most injuries occurred away from home and on roads. Table 2 summarily presents factors related to the mechanism of injury.

**TABLE 2:** Mechanisms of injury and related factors.

Variable	Frequency (%)
Injury mechanism (*N* = 393)	
Road traffic injury	275 (69.97)
Stab/cut wounds	47 (11.96)
Falls	27 (6.87)
Fights	20 (5.09)
Blunt trauma	24 (6.11)
Type of RTI (*N* = 275)	
Motorbike	144 (52.36)
Pedestrian	47 (17.09)
Passenger/private car	44 (16.00)
Animal	40 (14.55)
Place of occurrence (*N* = 322)	
Highway/road/paved road	197 (61.18)
Small, dirt road	47 (14.60)
Farm, field	24 (7.45)
Other (market, shopping center, other home)	21 (6.53)
Unknown	33 (10.25)
Means of transportation to hospital (*N* = 385)	
Motorbike	213 (55.32)
Private car	99 (25.71)
Police/law enforcement	30 (7.79)
Ambulance	24 (6.23)
Taxi	19 (4.94)
Sought care elsewhere before reaching the referral hospital (*N* = 438)	
Yes	68 (15.53)
No	370 (84.47)
Time interval between injury and arrival at hospital (*N* = 491)	
Within the same day	433 (88.19)
> 1–10 days	28 (5.70)

### 3.3. Clinical Parameters

**FIGURE 2:**
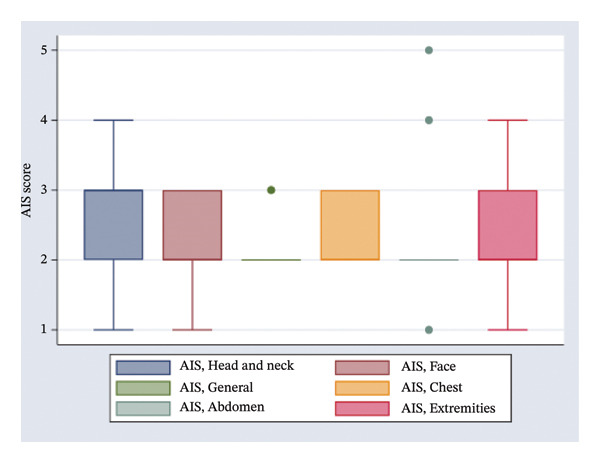
Distribution of the Abbreviated Injury Scores (AISs).

One hundred and forty‐two patients out of 296 (47.97%) had a pulse rate of less than 50/min on admission. The Glasgow Coma Scale was assessed for patients with confirmed or suspected brain injury upon admission: 164 patients out of 414 (39.62%) had a score of ≤ 8 (severe traumatic brain injury). An extremity fracture was the most frequent lesion in 192 out of 387 (49.61%) patients. Imaging was performed for 453 patients (88.64%). Table 3 summarizes the clinical parameters of the participants, whilst Figure 2 illustrates the distribution of the AIS per anatomic region.

**TABLE 3:** Clinical parameters of patients with injury.

Variable	Frequency (%)
Pulse rate on admission (*N* = 296)	
< 50/min	142 (47.97)
51–120/min	105 (35.47)
> 120/min	49 (16.55)
Glasgow Coma Scale (*N* = 414)	
≤ 8	164 (39.62)
9–15	250 (60.38)
Nature of injury (*N* = 387)	
Fracture (extremity)	65 (16.80)
Fracture (extremity) + head injury	24 (6.20)
Fracture (extremity) + head injury + abdominal injury	24 (6.20)
Fracture (extremity) + sprain/dislocation	79 (20.41)
Head injury (unique lesion)	9 (2.30)
Injury severity score (*N* = 511)	
< 15	448 (87.67)
≥ 15	63 (12.33)
Imaging studies performed (*N* = 453)	
Plain radiographs only	195 (43.05)
Plain radiographs + computed tomography	28 (6.18)
Computed tomography only	114 (25.17)

### 3.4. Delays in Obtaining CT

One hundred and thirty‐eight patients out of 439 (31.44%) obtained a CT within 24 h of hospital arrival or following request. Some difficulties in making payments for CT were reported for 42 out of 420 patients (10.00%). In the bivariate analysis, patients’ age, being male, lower educational achievement (that is, completion of primary and secondary school; OR = 2.8 [95%CI: 1.04–4.55], *p* = 0.038), having suffered an RTI, and reported difficulties paying for CT were independently associated with delays in obtaining CT. In the multivariate analysis, the patients’ age, male sex, reported difficulties paying for CT, and having suffered an RTI were predictors of delays in obtaining CT. Table 4 shows the predictors of delays in obtaining CT after modeling.

**TABLE 4:** Modeling of predictors of delay to obtain CT.

Variables	Bivariate analysis		Multivariate analysis^∗^	
	OR (95% CI)	*p* value	*a*OR (95% CI)	*p* value
Age group (years)				
18–24	2.22 (1.12–4.41)	0.023	4.51 (1.81–11.21)	0.001
25–29	3.16 (1.67–6.36)	0.001	2.23 (0.91–5.48)	0.079
30–34	3.05 (1.52–6.13)	0.002	2.82 (1.14–6.98)	0.025
35–39	3.96 (1.80–8.71)	0.001	7.59 (2.41–23.95)	0.001
≥ 40	1		1	
Sex				
Male	1.24 (0.82–1.87)	0.311	4.93 (2.27–10.71)	< 0.001
Female	1		1	
Difficulties paying for CT				
Yes	1.2 (0.67–5.55)	0.012	13.86 (4.93–38.94)	< 0.001
No	1		1	
Injury mechanism				
RTI	2.04 (1.24–3.34)	0.005	3.90 (1.92–7.91)	< 0.001
Others	1		1	

Abbreviations: aOR, adjusted odds ratio; OR: odds ratio.

^∗^Model with the lowest Akaike and Bayesian Information Criteria.

## 4. Discussion

The findings of this study indicate that nearly two‐thirds of patients with injuries in the study setting were male, with a mean age of 30 years. RTIs constituted about 70 per cent of all injuries, with slightly above half of these involving a motorbike. Motorbikes, the main means of commuting within the city, were also the principal means of transporting injured patients to the referral hospital. Most patients who sustained injuries arrived at the referral hospital on the same day as the injury, and fewer than a fifth reportedly sought care elsewhere before reaching the referral hospital.

Regarding the sex distribution, previous studies from Cameroon and other regions of the world have highlighted an increased risk of injury in males in general, with a corresponding increased risk of mortality [7, 8, 21, 22]. Even though some injury subtypes, such as domestic injuries, can be more prevalent amongst females, the major explanations for the male predilection of injury overall remain substance abuse, behaviors, jobs, and hobbies that increase the risk [4, 7]. The study findings further illustrate that RTIs remain the main mechanism of injury. Recently published data from a multicenter trauma registry in Cameroon indicated a similar trend [8], whilst other researchers had previously reported similar findings [7, 22]. It is noteworthy that the lack of a strongly regulated public transport system that prioritizes safety for all road users would continue to breed more cases of injuries, some of which are life‐threatening and others fatal. Furthermore, in the absence of an organized response system to injuries, especially regarding prehospital care and transportation to healthcare facilities, other methods have been improvised. Such improvised methods include motorbikes, private cars, and taxis, which are readily available and faster means of transporting injured patients in the study setting. These forms of transportation are usually not fit‐for‐purpose as they can potentially aggravate existing anatomic lesions.

Inasmuch as most of the injury patients were clinically stable upon admission, fractures, sprain, and/or dislocations at the extremities were the most frequent types of lesions. The first‐line imaging study requested to investigate these types of lesions remains conventional radiography. Ultrasonography use was not reported. The eFAST is a useful technique for assessing injury in emergency settings, as it helps depict haemothoraces and intra‐abdominal hemorrhage [23, 24] and is also useful for triaging patients who may require urgent surgery or further imaging. This finding highlights an educational opportunity for first‐line responders to improve the care that patients with injuries receive.

CT is the workhorse of hospital EDs, given its utility in assessing a wide range of emergency conditions. However, its use is associated with an increase in the overall direct cost of care [23]. In settings without national health insurance schemes for the general public, the use of such technology is through direct out‐of‐pocket (OOP) payments. OOP is a serious deterrent to the use of CT in resource‐poor settings, especially when much needed during emergency situations [11, 24]. Delays in obtaining imaging have been associated with increased hospital length of stay and early mortality in patients with emergency conditions, including injuries [12, 13, 23]. Factors independently associated with a delay in obtaining CT were young age, male sex, a lower educational achievement, reported difficulties in making payments for CT, and having suffered from an RTI. In the multivariate modeling, young age, being male, reporting financial difficulties, and having suffered an RTI were significant predictors of delays in obtaining CT.

The findings of this study consolidate understanding of injury epidemiology in Cameroon’s national territory, as RTIs remain the leading cause of injuries referred to the hospital [5, 25]. Reinforcing policies that enhance safety, such as mandatory wearing of helmets for motorbike riders and their passengers, mandatory use of car safety belts, control of alcohol consumption by drivers, formal training before obtaining a driver’s license for all categories of road users, and regular vehicle inspection, may curb the persistent trend of RTIs. In addition, implementing an efficient response system for the proper prehospital transportation of RTI victims to referral hospitals would minimize lesion aggravation and the time to seek healthcare. Given that imaging, especially CT, plays an important role in patient outcomes, further advocacy to policymakers is recommended to ensure that patients with injuries receive appropriate imaging when needed, without suffering significant delays due to a lack of financial support.

This study nevertheless has some limitations. First, data were collected from a single center, limiting its generalizability. In addition, some characteristics were assessed via self‐report, which may introduce reporting bias. Given that the data were provided by patients and, at times, their proxies, this could affect the accuracy of the information. Also, respondents did not provide data for some items (missing data). Furthermore, it is noteworthy that some patients with severe injuries probably never reached the referral hospital, and the findings, therefore, reflect only those who did. Despite these limitations, the study has some strengths. First, data collection was prospective, increasing objectivity. And although it is a single‐center study, this facility serves as a regional referral center, where moderate to severe cases are usually referred for management; therefore, it is ideal to capture most cases of injuries that require care beyond basic first aid.

## 5. Conclusions

The findings of this study indicate that, in this setting with a low HDI, RTIs, mainly from motorbike use, are the most common type of injury at the referral hospital. Males are predisposed and would often present with extremity fractures. Obtaining CT early in this setting was reportedly challenging, with the potential to affect the course of treatment and outcome. These findings highlight the need for, and the reinforcement of, measures to improve the quality of life of the population, protect all road users, and facilitate access to necessary emergency healthcare services. These services include relatively expensive technologies, such as CT, which plays a pivotal role in the management of injuries. We, therefore, advocate for sustained efforts to extend universal health coverage for such services.

**Nomenclature**
CT: Computed tomography
ED: Emergency department
RTI: Road traffic injury
AIS: Abbreviated injury score
ISS: Injury severity score
OOP: Out‐of‐pocket
OR: Odds ratio

## Author Contributions

Joshua Tambe conceived the study, supervised data collection, performed data analysis, and wrote the first draft of the manuscript. Yannick Onana participated in data collection, prepared the figures and tables, and proofread the manuscript. Nicholas Tendongfor reviewed the methods section and data analysis and corrected versions of the manuscript. Marie‐José Essi supervised data collection, proofread, and corrected versions of the manuscript. S. Ariane Christie and Alain Chichom‐Mefire participated equally as senior authors and corrected the final version of the manuscript. All authors reviewed the final version of the manuscript.

## Funding

Research reported in this publication was supported by the Fogarty International Center and Office of Strategic Coordination (OSC) of the National Institutes of Health under Award Number U54TW012087 to D‐SINE Africa.

## Disclosure

The content is solely the responsibility of the authors and does not necessarily represent the official views of the National Institutes of Health. The funder was not involved in the drafting, editing, or approval of the manuscript.

## Ethics Statement

This study was approved by the Institutional Review Board of the Faculty of Health Sciences, University of Buea (ethical clearance reference number: 2023/2058–03/UB/SG/IRB/FHS). Consent to participate in this study was obtained from the patient or legal proxy, and due to variations in literacy, this was either written (using standardized forms provided by the institutional review board) or verbal. This study was conducted in accordance with the ethical principles of the Declaration of Helsinki.

## Consent

Informed consent to collect data was provided for every participant. This also implied consent for publication as the data were anonymized.

## Conflicts of Interest

The authors declare no conflicts of interest.

## Data Availability

The dataset on which the conclusions of this study are based is available at Mendeley Data with doi: 10.17632/rkx9mr8x92.1.
